# Pulsatile Hormonal Signaling to Extracellular Signal-regulated Kinase: EXPLORING SYSTEM SENSITIVITY TO GONADOTROPIN-RELEASING HORMONE PULSE FREQUENCY AND WIDTH*

**DOI:** 10.1074/jbc.M113.532473

**Published:** 2014-01-30

**Authors:** Rebecca M. Perrett, Margaritis Voliotis, Stephen P. Armstrong, Robert C. Fowkes, George R. Pope, Krasimira Tsaneva-Atanasova, Craig A. McArdle

**Affiliations:** From the ‡ Laboratories for Integrative Neuroscience and Endocrinology, School of Clinical Sciences, University of Bristol, Bristol BS1 3NY, United Kingdom,; § School of Mathematics, University of Bristol, Bristol BS8 1TW, United Kingdom,; ¶ Endocrine Signaling Group, Royal Veterinary College, Royal College Street, London NW1 0TU, United Kingdom, and; ‖ Department of Mathematics, College of Engineering, Mathematics and Physical Sciences, University of Exeter, Exeter EX4 4QF United Kingdom

**Keywords:** Cell Signaling, ERK, MAP Kinases (MAPKs), Mathematical Modeling, Receptors, GnRH

## Abstract

Gonadotropin-releasing hormone (GnRH) is secreted in brief pulses that stimulate synthesis and secretion of pituitary gonadotropin hormones and thereby mediate control of reproduction. It acts via G-protein-coupled receptors to stimulate effectors, including ERK. Information could be encoded in GnRH pulse frequency, width, amplitude, or other features of pulse shape, but the relative importance of these features is unknown. Here we examine this using automated fluorescence microscopy and mathematical modeling, focusing on ERK signaling. The simplest scenario is one in which the system is linear, and response dynamics are relatively fast (compared with the signal dynamics). In this case integrated system output (ERK activation or ERK-driven transcription) will be roughly proportional to integrated input, but we find that this is not the case. Notably, we find that relatively slow response kinetics lead to ERK activity beyond the GnRH pulse, and this reduces sensitivity to pulse width. More generally, we show that the slowing of response kinetics through the signaling cascade creates a system that is robust to pulse width. We, therefore, show how various levels of response kinetics synergize to dictate system sensitivity to different features of pulsatile hormone input. We reveal the mathematical and biochemical basis of a dynamic GnRH signaling system that is robust to changes in pulse amplitude and width but is sensitive to changes in receptor occupancy and frequency, precisely the features that are tightly regulated and exploited to exert physiological control *in vivo*.

## Teaser

**Background:** Cellular decoding of stimulus dynamics is poorly understood.

**Results:** GnRH pulses activate ERK, and response kinetics determine sensitivity to different pulse features.

**Conclusion:** The system is sensitive to pulse frequency but robust to width; this distinction develops through the cascade and is dictated by response kinetics.

**Significance:** We describe mathematical and biochemical “design features” for pulsatile hormonal signaling.

## Introduction

Hormones typically exert concentration-dependent effects upon their target cells that can be described in terms of molecular mechanisms and can be modeled mathematically. However, many hormones are secreted in pulses, where information can be encoded in terms of pulse amplitude, frequency, width, or other features of pulse kinetics. Much less is known about the cellular responses to such dynamic inputs, and we are still lacking mathematical models of the underlying processes. Gonadotropin-releasing hormone (GnRH)^3^ is a neuropeptide hormone that stimulates the synthesis and secretion of luteinizing hormone (LH) and follicle-stimulating hormone (FSH) and thereby mediates central control of reproduction (1–3). It is secreted in pulses of a few minutes duration and acts via seven transmembrane receptors on pituitary gonadotropes to stimulate phospholipase C, mobilize Ca^2+^, and activate protein kinase C (PKC) isozymes. This activates mitogen-activated protein kinase (MAPK) pathways and Ca^2+^ effectors, and these in turn mediate the effects of GnRH on gonadotropin secretion and gene expression (1–5). GnRH pulse frequency varies under different physiological conditions, and pubertal increases in gonadotropin secretion and the pre-ovulatory gonadotropin surge are both driven by increases in GnRH pulse frequency (6, 7). GnRH effects are pulse frequency-dependent, with constant GnRH suppressing pituitary LH and FSH secretion, whereas GnRH pulses restore pulsatile gonadotropin secretion *in vivo* (8). Similarly, effects of GnRH pulses on LHβ, FSHβ, and GnRHR gene expression are maximal at submaximal frequency (6, 9–14). This is exploited therapeutically, as pulsatile administration of GnRH agonists can increase secretion of gonadotropins and gonadal steroids and thereby increase fertility (*e.g.* in ovulation induction during assisted reproduction), whereas sustained stimulation ultimately reduces steroid secretion, providing efficacy against steroid hormone-dependent cancers (2–4, 6, 7).

We have begun to explore mechanisms underlying dynamic GnRH signaling using semi-automated live cell imaging and mathematical modeling (15–17). Like many other seven-transmembrane receptors, the GnRHR activates the Raf/MEK/ERK cassette (1, 2, 4, 18, 19). On stimulation, extracellular-signal regulated kinases (ERKs) translocate to the nucleus where they phosphorylate transcription factors to control gene expression. GnRH activates ERKs 1 and 2, and ERKs can mediate GnRHR-stimulated transcription of the α-gonadotropin subunit (αGSU) as well as LHβ and FSHβ (1, 3, 4, 20). ERKs can mediate responses to pulsatile GnRH stimulation (21, 22), and the ERK cascade functions as a frequency decoder in other systems (23, 24). Using nuclear translocation of an ERK2-green fluorescent protein (GFP) reporter as a readout for ERK activation, we found that GnRH pulses cause rapid, transient, and reproducible ERK activation (16, 25), and we used these responses to fit an ordinary differential equation (ODE)-based model of GnRH signaling (17).

For pulsatile stimuli, information could be encoded by pulse frequency, amplitude, or by other features of pulse shape (26). In the simplest scenario the system is equally responsive to changes in pulse amplitude, frequency, or width. For example, when responses are rapid in onset and offset and adaptation does not occur, the system outputs can closely track hormone concentration so that there is a direct correlation between input and output at any point in time. This scenario predicts that integrated system output will be directly proportional to the integrated input; however, this is often not the case. For example, in *Caenorhabditis elegans* pulses of Ca^2+^ drive MAPK activation, and a recent study revealed a complex interplay between pulse frequency and duration in controlling sensory neuron MAPK activity (27). In this study the system was sensitive to changes in pulse frequency but robust to changes in pulse width. To our knowledge similar issues have not been explored for GnRHR signaling. Indeed, numerous experiments have focused on the cellular decoding of GnRH pulse frequency (6–17), but the relevance of other pulse features is essentially unknown.

Here we consider this using empirical and mathematical approaches and focusing on the ERK pathway. We find that it does not follow the simple integrative tracking scenario above, first, because of the nonlinear relationship between GnRH concentration and receptor occupancy and, second, because relatively slow response kinetics cause signaling beyond the GnRH pulse. This creates a dynamic system that is robust to changes in pulse amplitude and width but is sensitive to changes in receptor occupancy and frequency, precisely the features that are regulated for physiological control *in vivo*. More generally, we find that the slowing of response kinetics through the signal-transduction cascade creates robustness to pulse width. Indeed, upstream and downstream outputs in the cascade are shown to be sensitive to pulse frequency, whereas upstream outputs are more sensitive to pulse width than downstream ones. This implies that when cells receive a series of stimulatory pulses, the information that could be conveyed by these distinct features (pulse width and interval) depends on response kinetics and the system output being measured.

## EXPERIMENTAL PROCEDURES

### 

#### 

##### Vectors and Cell Culture

HeLa cells (European Collection of Cell Cultures (ECACC)) were cultured in Costar black-wall 96-well plates (Corning, Arlington, UK) using 10% FCS-supplemented Dulbecco's modified Eagle's medium (DMEM). In cells transduced with ERK2-GFP, endogenous ERKs were first knocked down by transfection with 1 nm siRNA targeting noncoding regions of ERK1 and ERK2 to prevent overexpression as described (28, 29). Cells were transduced in DMEM with 2% FCS 24 h after plating and siRNA knockdown using Ad ERK2-GFP (2 pfu/nl), Ad NLS-BFP (75 pfu/nl), Ad GnRHR (1 pfu/nl unless otherwise stated), or Ad Egr-1-luc (1 pfu/nl) as described (15, 30). Ad vectors were grown to high titer and purified according to standard protocols (30). The Ad-containing medium was removed after 4–6 h and replaced with DMEM containing 0.1% FCS. The cells were then cultured for 16–24 h before GnRH stimulation. For some experiments we explored signaling via endogenous ERK as described (28, 29). Hereafter we use the term ERK to mean ERK1 and/or ERK2 and provide the numerical designation only when a specific form is meant (as in ERK2-GFP). For some experiments we explored signaling via the endogenous murine GnRHR of the gonadotrope-derived LβT2 cell line (kindly provided by Prof. P. Mellon, University of San Diego, CA). These were cultured in Costar black-wall 96-well plates coated with Matrigel (BD Bioscience) and were also incubated in DMEM containing 0.1% FCS for 16–24 h before GnRH stimulation as described (4, 16, 29).

##### Image Acquisition and Analysis

Imaging was with an InCell Analyzer 1000 (GE Healthcare) high content imaging platform with 1–3 fields of view (0.6 mm^2^) and a 10× objective (29). Treatments were in duplicate or triplicate wells, and each field typically contained 300–500 cells. For live cell imaging, cells were plated in 96-well plates at 5 × 10^3^ cells/well and cultured as above. Medium was replaced 25 min before imaging with phenol red-free DMEM/F-12 (with 100 μg/ml BSA and 10 μg/ml apotransferrin) and, if Ad NLS-BFP was not included, contained 400 nm Hoechst nuclear stain. Cells were imaged at 37 °C in a 5% CO_2_ humidified atmosphere and stimulated with GnRH pulses (5-min pulses, terminated by washing) at the indicated frequency. For fixed cell imaging, cells were plated in 96-well plates and cultured as above. After treatment, they were washed in ice-cold phosphate-buffered saline (PBS), fixed with 4% paraformaldehyde, then stained for ppERK and/or ERK and nuclei (DAPI) before being imaged as described (28, 29). Image analysis was with InCell Analyzer Work station 3.5 software (IN Cell Investigator, GE Healthcare). Green channel (GFP and ERK) and blue channel (BFP, Hoechst, and DAPI) images were used to define whole-cell and nuclear regions (respectively), and ppERK was quantified in the red channel.

##### Luciferase Assays

Cells were plated and transduced with Ad expressing an Egr-1 promoter luciferase reporter (Ad Egr-1 luc) and Ad GnRHR (30). After treatment, as detailed in the figure legends, they were washed in ice-cold PBS and lysed, and luciferase activity was determined as described (28, 29). Data are reported as relative light units normalized as -fold change over control.

##### Mathematical Modeling

We have previously described a model for GnRH signaling to the transcriptome via ERK and NFAT that is based on a system of 35 ODEs and mass action kinetics (17). This is unnecessarily complex for the current study (in which we do not consider NFAT signaling), so we simplified it by removing equations describing the Ca^2+^/calmodulin/calcineurin/NFAT pathway. We then used a genetic fitting algorithm to train the model against previously published data for ERK2-GFP translocation in response to sustained or pulsatile GnRH at a range of GnRH concentrations (Figs. 2 and 3 in Armstrong *et al.* (16)). The simplified model, best-fit parameters, and details of the genetic algorithm are given below, and the model parameters are in Table 1.

We model the GnRH input, denoted [GnRH], using a square wave, expressed in terms of a Heaviside step function, *H*, in the following way,

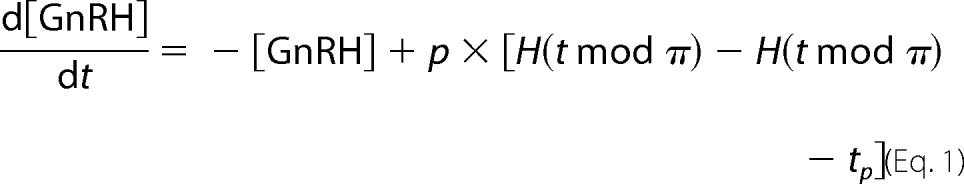
 where *p* is the pulse magnitude, Π is the pulse period, *t_p_* is the pulse duration (width), and mod is the modulo operation. The first step in our model is binding of GnRH to its receptor. Subsequently, the hormone bound receptor complex ([HR]) reacts with a G protein ([GQ]) to produce an effector ([E]) that promotes the activation of ERK by activating the ERK kinase (MEK). In our model ERK is allowed to shuttle between the nucleus and the cytoplasm. Activation (phosphorylation) of ERK occurs only at the cytoplasm and is modeled as in Markevich *et al.* (31). On the other hand, deactivation (de-phosphorylation) of ERK occurs in both cellular compartments (*i.e.* nucleus and cytoplasm). The model also incorporates a negative feedback mechanism through which activated cytosolic ERK ([ppE_C_]) suppresses the levels of the effector [E]. Finally, active nuclear ERK ([ppE_n_]) drives the activation of a transcription factor ([TF1], such as early growth response factor 1 (Egr-1)), which in turn increases expression of TF1DT (TF1-dependent transcript). The above are mathematically represented by the following system of equations.

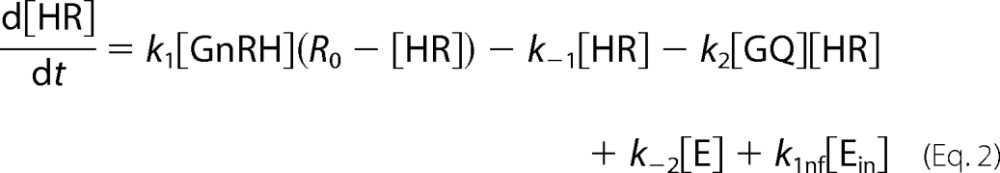


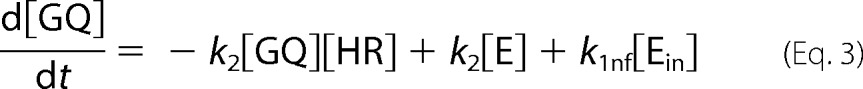


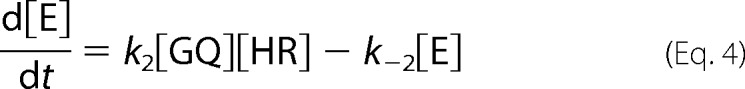


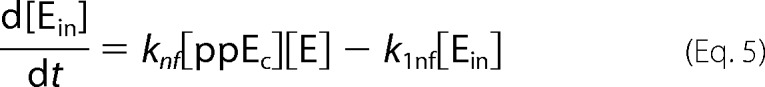


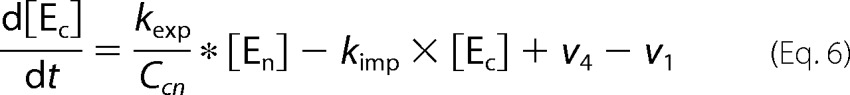


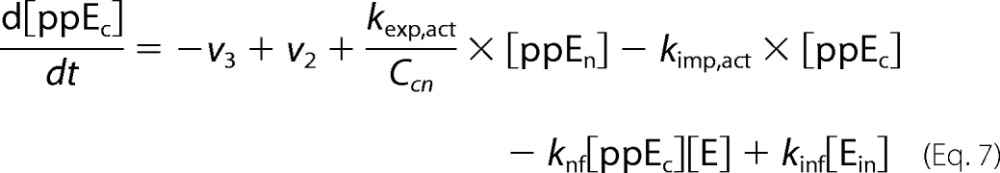


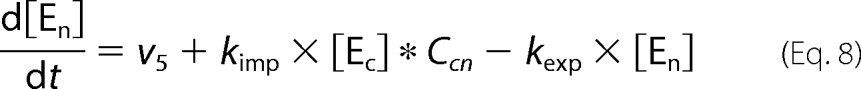


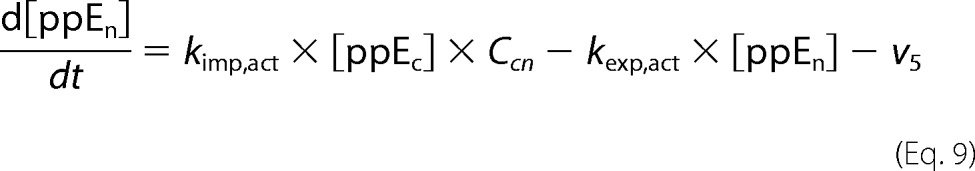


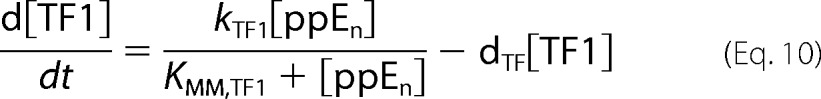


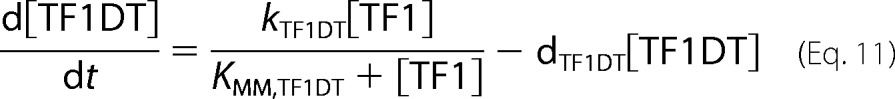
 Fluxes *v*_1_ − *v*_5_ are defined as in Cullen and Lockyer (23),

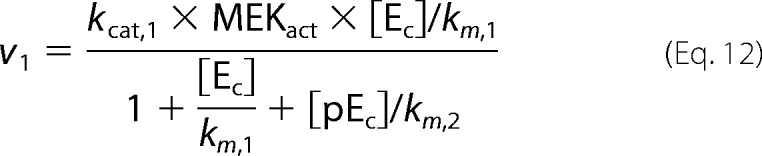


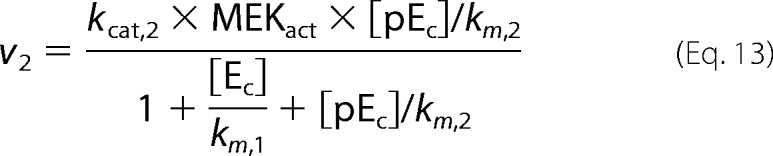


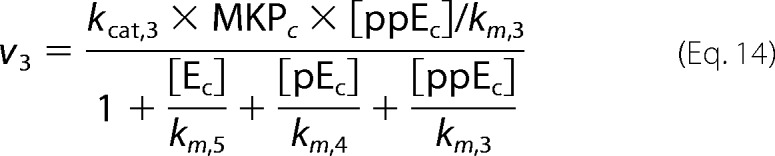


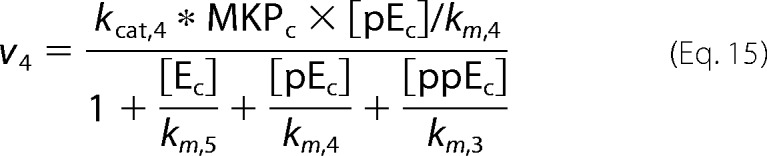


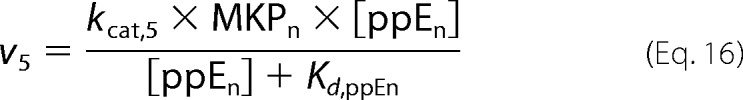
 where we have assumed that MEK (ERK kinase activated by effector E) is well approximated by the Michaelis-Menten equation,

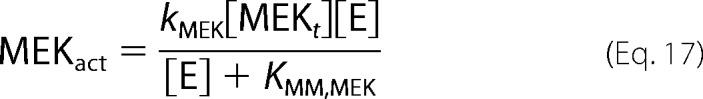
 We fitted the model parameters to reproduce (i) our experimental measurements of ERK activation dynamics for 1 min, 10 min, and constant GnRH stimulation and (ii) dose-response curves for ERK activation and for a downstream reporter gene. Fitting was performed using a genetic algorithm. A population of candidate parameter vectors was initialized using the parameters values found in Tsaneva-Atanasova *et al.* (17). At the beginning of each iteration (generation) each parameter marked with an asterisk in Table 1 was randomly perturbed (θ_new_ − θ_current_ (1 + 0.1ϵ) where ϵ is drawn from standard normal distribution) for all vectors in the population. The mutated vectors were then used to simulate the model, and their goodness of fit was assessed in terms of the mean square error from the experimental objectives. After each iteration a new population was created by sampling (with replacement) vectors from the current population with probability proportional to their ranking.

## RESULTS

**FIGURE 1.**
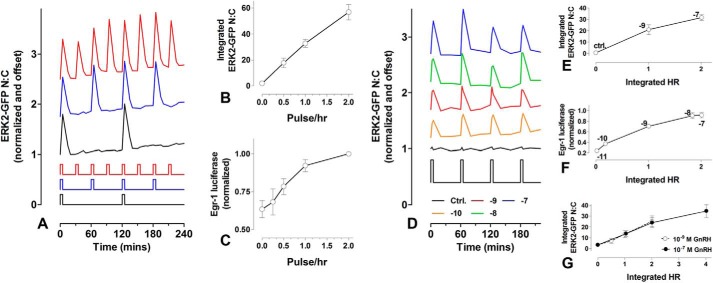
**Effects of pulsatile GnRH on ERK2-GFP localization and Egr-1 promoter activity.** *Panel A*, data for HeLa cells transfected with siRNA to knock-down endogenous ERK1/2 and transduced with Ad GnRHR and Ad ERK2-GFP for live cell imaging. Cells received 5-min pulses of 10^−7^ m GnRH at 30 min (*red*), 60 min (*blue*), and 120 min (*black*) intervals as indicated by the *bars*. GnRH pulses were terminated by washing. Fluorescence microscopy was used for imaging and for calculation of the N:C ERK2-GFP ratios. These were normalized to the value at time 0 and are offset on the *y* axis for clarity (*blue*, +0.8, *red*, +1.6). The integrated ERK2-GFP response (calculated after subtraction of basal values) is plotted in *panel B* against GnRH pulse frequency. *Panel C*, data from HeLa cells transduced with Ad Egr-1 luciferase and Ad GnRHR and stimulated for 8 h with 5-min pulses of 10^−7^ m GnRH at 30-, 60-, 120-, or 240-min intervals before luciferase measurement. The data are normalized to the response with the highest GnRH pulse frequency. *Panels D*, cells treated as for *panel A* except that they received 0 (control, *black*), 10^−10^ m (*orange*), 10^−9^ m (*red*), 10^−8^ m (*green*), or 10^−7^ m (*blue*) GnRH pulses of 5-min width at 60-min intervals. N:C ERK2-GFP was normalized to the value at time 0 and are offset on the *y* axis for clarity (*orange*, +0.4, *red*, +0.8; *green*, +1.2; *blue*, +1.6). The integrated ERK2-GFP response was calculated for a series of experiments (fewer GnRH concentrations, log M [GnRH] on graph), and is plotted in *panel E* against integrated hormone receptor occupancy (*HR*) estimated for each GnRH concentration (using the model under “Experimental Procedures” and Table 1). *Panel F*, data from HeLa cells receiving Ad Egr-1 luciferase and Ad GnRHR and stimulated 8 h with 5-min pulses of GnRH at 60-min intervals using 10^−11^-10^−7^ m. Luciferase activity is plotted against integrated HR. The data are normalized to highest response in each experiment (log M [GnRH] indicated on graph). *Panel G*, data from live cell experiments similar to *panel F* except that only two GnRH concentrations were used (10^−9^ and 10^−7^ m), and Ad GnRHR titer was varied (0.3, 1, and 3 pfu/nl) to provide cell surface GnRHR expression at ∼40,000, 80,000, and 160,000 sites/cell. For each GnRH concentration the integrated N:C ERK2-GFP translocation response is plotted against integrated HR. The data shown are either from single representative experiments (*A* and *D*) or are pooled from three separate experiments (mean ± S.E., *n* = 3) with duplicate or triplicate wells in each.

Nuclear translocation of ERK2-GFP can be measured by calculation of the nuclear:cytoplasmic ratio (N:C), providing a live-cell readout for GnRHR-mediated ERK activation. Using this assay we previously showed that GnRH pulses cause rapid and transient ERK activation without adaptation from pulse to pulse (16), which is indicative of the simple integrative tracking scenario described above. Here we performed similar experiments confirming the occurrence of rapid, transient, and reproducible ERK2-GFP responses in cells stimulated with 5-min pulses of 10^−7^ m GnRH at 30-, 60-, or 120-min intervals (Fig. 1*A*). Because the responses were comparable to each sequential pulse, the area under the curve (integrated ERK2-GFP N:C) was approximately linearly dependent on GnRH pulse frequency (and, therefore, to the integrated square wave input) (Fig. 1*B*). As a readout for ERK-driven transcription, we also measured Egr-1 luciferase activity (cells stimulated for 8 h with 5-min pulses of 10^−7^ m GnRH at 30-, 60-, 120-, or 240-min intervals), and similarly, a near linear relationship between pulse frequency and response was seen (Fig. 1*C*, see also Armstrong *et al.* (16)). These data are consistent with the ERK pathway acting as a simple integrative tracker of GnRH concentration, but an obvious concern is that the relationship between hormone concentration and receptor occupancy is not linear (being dictated by Michaelis-Menten kinetics) such that integrated GnRHR occupancy (HR) might provide a more meaningful system input. When we tested this by calculating integrated HR for ERK2-GFP imaging experiments with GnRH at a fixed pulse interval (60 min) and varied concentration (0 or 10^−10^-10^−7^ m), each GnRH pulse caused rapid and transient translocation of ERK2-GFP to the nucleus, effects that were both concentration-dependent and reproducible (Fig. 1*D*, see also Armstrong *et al.* (16)). The integrated ERK2-GFP N:C response was not directly proportional to GnRH concentration (increasing only 33% as GnRH concentration was increased 100-fold from 10^−9^ to 10^−7^ m; not shown) but was approximately linearly dependent on integrated HR (estimated using the ODE-based model described under “Experimental Procedures” and Table 1) (Fig. 1*E*). We also measured Egr-1 luciferase activity (stimulation for 8 h with 5min pulses of 10^−11^-10^−7^ m GnRH at 60-min intervals), and this also revealed a near linear relationship between integrated HR and response (Fig. 1*F*). As an alternative approach we varied integrated HR by varying Ad GnRHR titer to express cell surface GnRHR at 40,000, 80,000, and 160,000 sites/cell. The cells were again stimulated with 5-min pulses of GnRH (10^−9^ or 10^−7^ m) at 60-min intervals, and for each GnRH concentration the integrated ERK2-GFP response was plotted against the integrated HR. This revealed near linear input-output relationships that were essentially superimposable for the two GnRH concentrations (Fig. 1*G*).

**TABLE 1:** **Model parameters**

Symbol	Description	Value
*R*_0_	Total GnRH receptor concentration	0.1 μm*^a^*
*k*_1_	Reaction rate constants for binding between GnRH and its receptor	5000 μm^−1^ min^−1^
*k*_−1_	Reaction rate constants for unbinding of GnRH from its receptor	5 min^−1^
*k*_2_	Reaction rate constants for binding between HR and GQ	5 μm^−1^ min^−1^*^a^*
*k*_−2_	Reaction rate constants for unbinding of GQ from HR	1 s^−1^*^a^*
*k*_3_	reaction rate constants for binding between E and ppE_n_	5.5 μm^−1^ min^−1^
*k*_−3_	Reaction rate constants for deactivation of effector E	0.05 min^−1^
*k*_exp,act_	Reaction rate constants for export of ppE_n_ from the nucleus	0.75 min^−1^*^a^*
*k*_exp_	Reaction rate constants for export of E_n_ from the nucleus	0.75 min^−1^*^a^*
*k*_imp.act_	Reaction rate constants for import of ppE_c_ into the nucleus	0.4 min^−1^*^a^*
*k*_imp_	Reaction rate constants for import of E_c_ into the nucleus	0.2 min^−1^*^a^*
*k*_mek_	Catalysis rate constant for MEK activation by E	10 min^−1^*^a^*
*K*_MM,MEK_	Michaelis-Menten constant for MEK activation	0.05 μm*^a^*
ERK_tot_	Total ERK concentration	0.9 μm
MEK_tot_	Cytosolic MEK concentration	0.6 μm*^a^*
*k*_MEK,basal_	Rate constant for basal MEK activation	0
*k*_cat,1_	Parameter in flux *v*_1_	0.6 min^−1^
*k*_cat,2_	Parameter in flux *v*_2_	5 min^−1^
*k*_cat,3_	Parameter in flux *v*_3_	4 min^−1^
*k*_cat,4_	Parameter in flux *v*_4_	4 min^−1^
*k*_cat,5_	Parameter in flux *v*_5_	4 min^−1^
*k_m_*_,1_	Parameter in fluxes *v*_1_–*v*_2_	0.05 μm
*k_m_*_,2_	Parameter in fluxes *v*_1_–*v*_2_	0.0339 μm
*k_m_*_,3_	Parameter in fluxes *v*_3_–*v*_4_	0.022 μm
*k_m_*_,4_	Parameter in fluxes *v*_3_–*v*_4_	0.0180 μm
*k_m_*_,5_	Parameter in fluxes *v*_3_–*v*_4_	0.0782 μm
*k_d,_*_ppEn_	Parameter in fluxes *v*_5_	0.01 μm
MKP_n_	Nuclear phosphatase concentration	0.05 μm
MKP_c_	Cytosolic phosphatase concentration	0.05 μm
*C_cn_*	Nucleus to cytoplasm volume ratio	3
*d*_TF1_	Degradation rate constant of the TF1	0.023 min^−1^
*k*_TF1_	Rate constant for TF1 activation	0.03 μm min^−1^
*K*_MM,TF1_	Michaelis-Menten constant for TF1 activation	0.4 μm
*d*_TF1DT_	Degradation rate constant of TF1DT	0.05 min^−1^
*k*_TF1DT_	Rate constant for TF1DT activation	0.03 μm min^−1^
*K*_MM,TF1DT_	Michaelis-Menten constant for TF1DT activation	0.5 μm

*^a^* Parameter were fitted to our experimental data as described under “Experimental Procedures.”

**FIGURE 2.**
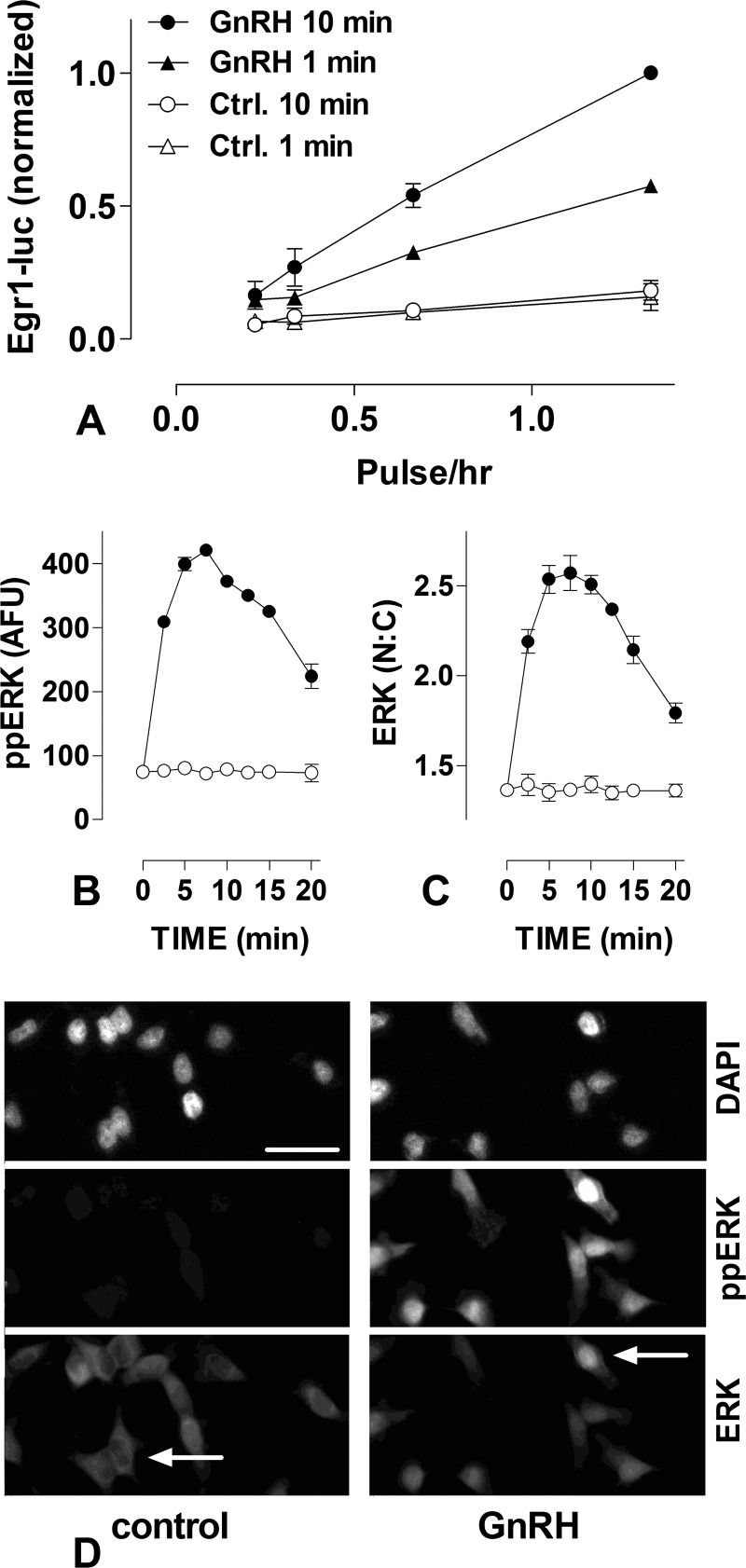
**Effects of pulsatile GnRH on Egr-1 promoter activity and ERK activation; varied GnRH stimulus duration.** *Panel A*, Ad GnRHR and Ad Egr1-luc transduced cells were stimulated for 6 h with pulses of 0 (control) or 10^−7^ m GnRH at 45-, 90-, 180-, and 360-min intervals. Pulses (1 or 10 min) were terminated by washing. Luciferase activity from 3 experiments is shown pooled after normalization to the 10-min width/45-min interval data (mean ± S.E., *n* = 3). *Panels B* and *C*, cells transduced with Ad GnRHR were stimulated for 0 or 2.5–20 min with 0 (*open circles*) or 10^−7^ m (*filled circles*) GnRH then stained for ppERK, ERK, and DAPI, which were quantified as described under “Experimental Procedures.” The data shown are from a single representative experiment with whole cell ppERK values reported in arbitrary fluorescence units (*AFU*) after background subtraction. Nuclear and cytoplasmic ERK expression was also determined, and these values (150–650 arbitrary fluorescence units after background subtraction) were used to calculate the N:C ratio shown. Similar experiments were undertaken with cells transfected with ERK siRNA and transduced with Ad GnRHR and Ad ERK2-GFP before being stimulated for 0 or 2–14 min with 10^−7^ m GnRH and then stained for ppERK2. This revealed very similar response kinetics (data not shown, maximal increase of ∼2.5-fold occurring at 6 min), and data pooled from 3 such experiments revealed that the integrated ppERK2 response to 1 min was 6% that of the response to 10 min. *Panel D* shows representative images from cells stimulated for 7.5 min with 0 (control) or 10^−7^ m GnRH before fixation and staining for DAPI, ppERK, and ERK, as indicated. Note that the increase in whole cell ppERK (*middle panels*) and the associated ERK translocation to the nucleus (*arrows* in *lower panels*). These representative images show ∼2% of the area actually imaged to generate the *x-y* plots in *panels B* and C. *Scale bar*, ∼40 μm.

The data above are compatible with ERK acting as a simple integrative tracker of GnRHR occupancy but not of GnRH concentration. If so, the system would be equally sensitive to variation in GnRH pulse frequency and width, but we found that this is not the case. We measured Egr-1 luciferase activity after stimulating cells for 6 h with 1- or 10-min pulses of 10^−7^ m GnRH at varied pulse frequency (control cells were pulsed with normal medium (no GnRH) using the same pulse frequency and width). As shown (Fig. 2*A*), there were near linear relationships between pulse frequency and the transcriptional response, whereas increasing pulse width from 1 to 10 min caused only a doubling of the output.

A possible explanation for the observed system robustness to GnRH pulse width is that the ppERK activation response is rapid and transient with pronounced activation occurring in the first minute of stimulation, but we found that this is not the case. As shown (Fig. 2, *B–D*) 10^−7^ m GnRH increased both ppERK levels and the N:C ratio for ERK. Both effects increased to maxima at 5–10 min and reduced thereafter. Similarly, when the ERK knockdown/ERK2-GFP add-back protocol was used, ppERK2 levels increased to a maximum at 6 min (in cells stimulated continuously with 10^−7^ m GnRH) and reduced gradually thereafter so that the integrated response to 1 min was actually <2% of the 10-min response (not shown). Thus, if the integrated response during the GnRH pulse alone dictated the transcriptional output, we would expect extreme sensitivity to pulse width rather than the robustness observed (Fig. 2*A*).

**FIGURE 3.**
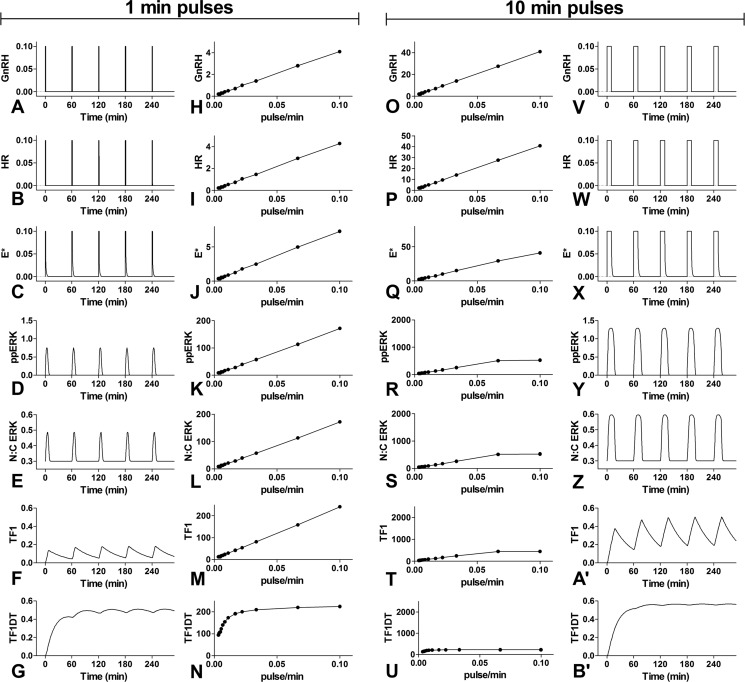
**Mathematical modeling of GnRHR-mediated ERK signaling at varied pulse frequency.** A mathematical ODE-based model (“Experimental Procedures” and Table 1) was implemented in Matlab to predict response time-courses in cells receiving 1- or 10-min pulses of 10^−7^ m GnRH. The measures shown (working down the cascade and page) are GnRH concentration (*GnRH*)-, GnRHR occupancy (*HR*)-, upstream effector activation (E*)-, whole cell dual phosphorylated ERK (*ppERK*)-, N:C ERK ratio (*N:C ERK*)-, activated transcription factor 1 (*TF1*)-, and transcription factor 1-dependent transcript (*TF1DT*). The *left panels* (*A–G*) show time-courses with 1-min GnRH pulses at 60-min intervals. Similar modeling was performed with 1-min GnRH pulses and varied frequencies and used for calculation of areas under the curves. These integrals are plotted against frequency in the *second column*. Note that pulse frequency is proportional to input integral, so these are essentially integrated input-output relationships matched for pulse width and measure. The *right panels* (*V–B*′) show time courses with 10-min pulses of GnRH at 60-min intervals. Again, similar modeling was performed for calculation of integrated input-output relationships, as shown in the third column. Here, maximal stimulation is actually constant activation (10-min pulses every 10 min). Note that the *y* axis ranges for column 3 are 10 times higher than those for column 2 so that, if the system was equally sensitive to pulse frequency and width, the plots in columns 2 and 3 would appear similar.

We used mathematical modeling to explore this in more detail. We reduced our published model (17) by removing equations describing GnRH signaling via the Ca^2+^ and NFAT pathway and obtained parameter values using a genetic algorithm to fit the model to published ERK2-GFP translocation data (Experimental Procedures and Table 1). We then simulated signaling with pulsatile GnRH. The *left panels* of Fig. 3 show data predicted for 1-min GnRH pulses at 60-min intervals after the signaling cascade from the input (GnRH concentration; Fig. 3*A*) to receptor occupancy (*HR*, Fig. 3*B*), upstream effector activation (E*, Fig. 3*C*), ERK activation (*ppERK*, Fig. 3*D*), nuclear translocation of ERK (*N:C ERK*, Fig. 3*E*), activation of an ERK-dependent transcription factor (*TF1*, Fig. 3*F*), and cellular levels of a TF1-dependent transcript (*TF1DT*, Fig. 3*G*). This simulation demonstrates that slow response dynamics lead to more sluggish responses when moving down the pathway (*i.e.* the broadening of responses from Fig. 3, *A–F*), giving rise to responses that do not return to prestimulation values between pulses and cumulative or saw-tooth responses for the most downstream measures (Fig. 3, *F* and *G*). Similar analysis was performed for varied pulse frequency, and the predicted time-courses were used to calculate areas under the curve for each of these predicted measures. These were plotted against GnRH pulse frequency (Fig. 3, *H–N*) and show linear or near linear relationships between pulse frequency and integrated GnRH input (Fig. 3*H*) and for the upstream signals that closely track the input (Fig. 3, *I–L*). However, the relationship becomes nonlinear for the downstream readouts (notably for *TF1DT*, Fig. 3*N*), revealing how slow response dynamics can increase the efficiency of signaling. For example, a pulse interval of 45 min is predicted to give near maximal transcriptional response (TF1DT 90% of maximum) despite a much lower activation of the upstream effector (E* 20% of maximum).

**FIGURE 4.**
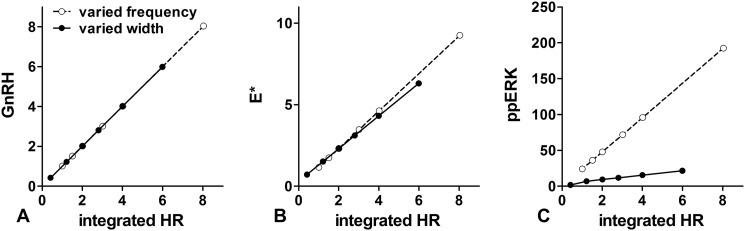
**Mathematical modeling of GnRHR-mediated ERK signaling at varied receptor occupancy.** The mathematical ODE-based model was implemented in Matlab to predict time-courses of responses in cells treated with 10^−7^ m GnRH as described under Fig. 4, except that a broader range of pulse widths was tested. Predicted values for integrated GnRH, E*, or ppERK are plotted against integrated HR. For some runs pulse width was fixed (5 min) and integrated HR was varied by varying pulse interval; for others pulse interval was fixed (60 min), and integrated HR was varied by varying pulse width, as indicated. If the system was equally sensitive to pulse frequency and pulse width, the two plots (varied frequency and varied width) would be superimposable. This is (inevitably) the case for GnRH but the curves separate, moving down the cascade to E* and then further from E* to ppERK.

The 10-min pulse data (Fig. 3*V*-B′) again revealed more sluggish responses moving down the cascade. Varying pulse intervals and plotting integrated measures against pulse frequency (Fig. 3, *O–U*) also revealed near linear relationships upstream (*i.e.* HR and E* in Fig. 3, *P* and *Q*) and nonlinear responses moving downstream toward TF1DT (Fig. 3*U*). To aid visualization we set the *y* axis ranges for column 3 10× higher than for column 2. If the system was equally sensitive to pulse width and frequency, the plots in columns 2 and 3 would appear similar, but this was not always the case. For the upstream measures (GnRH in Fig. 3, *H* and *O*, E* in Fig. 3, *I* and *P*) the response curves have similar steepness (*i.e.* the gradient is 10× higher for 10-min pulses than for 1-min pulses), but moving down the pathway the response curves are less steep for 10-min pulses. As an alternative illustration we plot 3 integrated measures (GnRH, E*, and ppERK) against integrated HR for simulations where HR was controlled by varying pulse frequency or width. As shown (Fig. 4) the relationships between integrated HR and integrated GnRH or E* are linear and are largely superimposable for both conditions (because HR and E* track GnRH so closely over time). In contrast, the gradient of the integrated ppERK/integrated HR response curve is much higher when HR is controlled by varying pulse frequency than it is for varied pulse width. Thus, the modeling reveals a pathway in which upstream responses provide simple integrative tracking of input (as revealed by similar sensitivity to pulse frequency and duration) but for which slow response dynamics cause more complex behavior (*i.e.* relative insensitivity to pulse width) further downstream.

**FIGURE 5.**
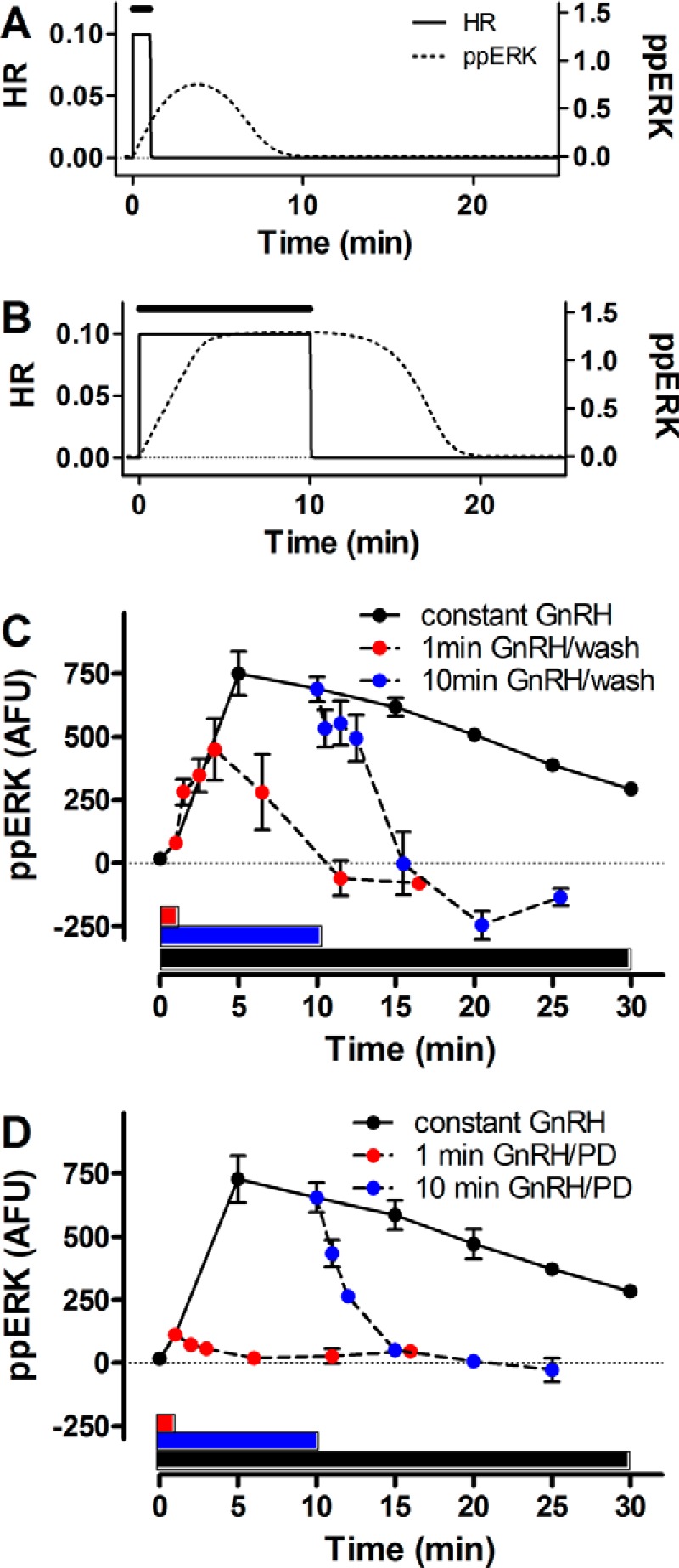
**ERK activation continues beyond the GnRH pulse in HeLa cells.** *Panels A* and *B* show predicted HR (*solid line*) and ppERK (*dotted line*) values for 1 min (*A*) or 10 min (*B*) of stimulation with 10^−7^ m GnRH (*horizontal bars*). These were modeled as described under Fig. 4 and are the same data as the first pulse in Fig. 4, *A*, *B*, *D*, *V*, *W*, and *Y* (but with an expanded scale)), illustrating continuation of ERK activity beyond the GnRH pulse. *Panel C* shows empirical data testing for such activity. Ad GnRHR transduced cells were stimulated for up to 30 min with 10^−7^ m GnRH (*black circles*) or were washed to remove GnRH after 1 min (*red circles*) or 10 min (*blue circles*), then incubated further in normal medium before measurement of whole cell ppERK levels. *Bars* of corresponding color indicate periods of GnRH exposure (1, 10, and 30 min). ppERK values in control cells (that received exactly the same manipulations but without GnRH) have been subtracted (mean ± S.E., *n* = 3). *Panel D* shows data from a parallel series of experiments in which the 1- and 10-min GnRH pulses were terminated by the addition of the PD184352 MEK inhibitor at 10 μm (mean ± S.E., *n* = 3). *AFU*, arbitrary fluorescence units.

**FIGURE 6.**
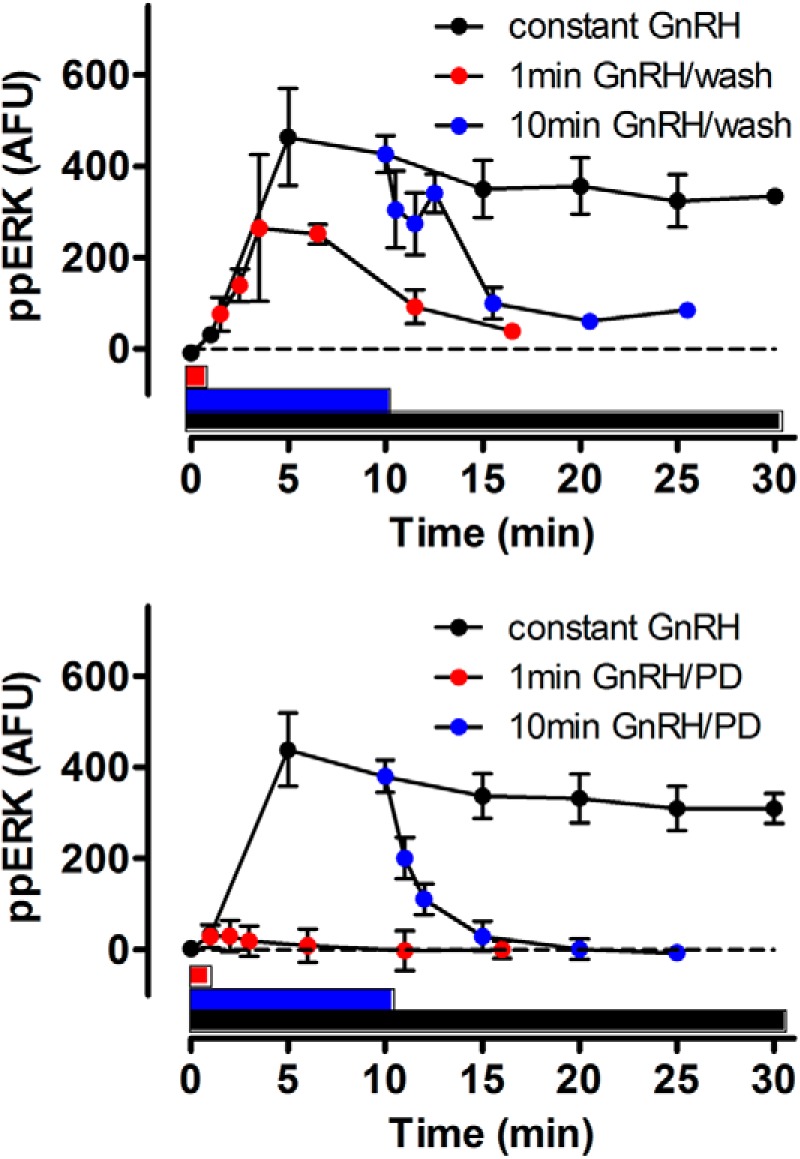
**ERK activation continues beyond the GnRH pulse in LβT2 cells.** *Upper panel*, cells grown on Matrigel in 96-well plates were stimulated for up to 30 min with 10^−7^ m GnRH (*black circles*) or were washed to remove GnRH after 1 min (*red circles*) or 10 min (*blue circles*) and then incubated further in normal medium before measurement of whole cell ppERK levels. The *horizontal bars* of corresponding color indicate the periods of GnRH exposure (1, 10, and 30 min). ppERK values in control cells (that received exactly the same manipulations but without GnRH) have been subtracted (mean ± S.E., *n* = 3). *Lower panel*, data from a parallel series of experiments in which the 1- and 10-min GnRH pulses were terminated by the addition of the PD184352 MEK inhibitor at 10 μm (mean ± S.E., *n* = 3). *AFU*, arbitrary fluorescence units.

Fig. 4*C* illustrates the model prediction of greater system sensitivity to pulse frequency than to pulse width, precisely as observed experimentally (Fig. 2*A*). To explore this we considered response kinetics in more detail for single pulses of 1- or 10-min duration. The model predicted continuation of ERK activation for a short period after the GnRH pulse, an effect that was particularly pronounced for the shorter GnRH pulse, with ppERK levels actually predicted to rise after pulse termination (Fig. 5*A*). The obvious consequence of this is that the integrated ppERK response for a 1-min GnRH pulse is much greater than 10% of the response for a 10-min pulse, providing a possible explanation for relative insensitivity of the system to pulse width (Fig. 2*A* and 5). To test this we measured the 30-min time-courses for ppERK levels in cells that were either continuously stimulated with GnRH or for which GnRH was removed (by 5 rapid washes) after 1 or 10 min of stimulation. Control cells received identical treatments but without GnRH, and control values were subtracted to illustrate the GnRH effect alone. As shown in Fig. 5*C*, GnRH caused the expected activation of ERK, with ppERK levels increasing to a maximum at 5 min and reducing gradually thereafter. ppERK levels reduced more rapidly when GnRH was removed after 10 min, returning to near basal values at 15 min. However, when GnRH was removed after 1 min of stimulation, ppERK levels continued to rise, reaching a maximum at 5 min and then reducing toward the basal level at 10–15 min. Thus, the majority (>90%) of the ppERK response occurs after the 1-min GnRH pulse. In parallel experiments the 1- and 10-min pulses were terminated by the addition of a MEK inhibitor (PD184352), and this rapidly reversed the ppERK responses (Fig. 5*D*). Notably, MEK inhibitor addition 1 min after GnRH caused an immediate reduction in ppERK levels and prevented the post-pulse rise in ppERK seen with the wash protocol (Fig. 5, *C* and *D*). Very similar data were obtained when these experiments were repeated using a gonadotrope-derived cell line that expresses endogenous GnRHR (Fig. 6). In both models, for the 1-min pulse, the majority of the ppERK response actually occurs after the GnRH pulse and is dependent upon ongoing MEK activity, implying that MEK is slowly inactivated after the GnRH pulse (Figs. 5 and 6).

**FIGURE 7.**
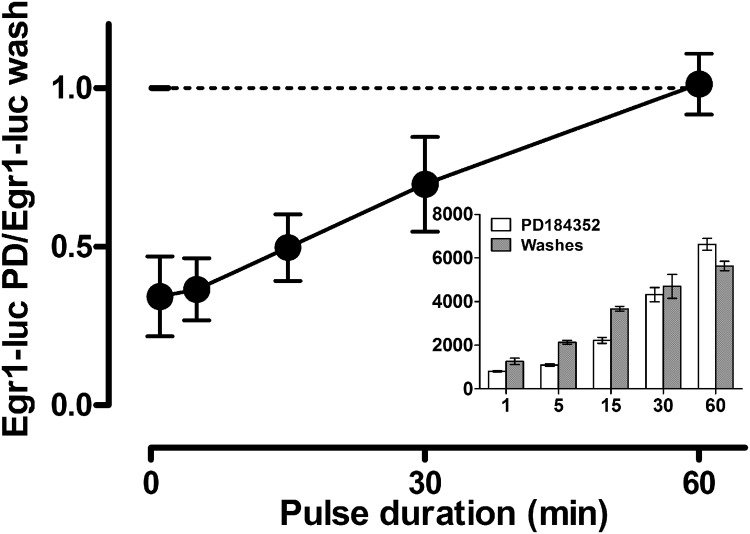
**Effects of a single GnRH pulse on Egr-1 promoter activity.** Ad GnRHR- and Ad Egr1-luc-transduced cells were stimulated with 10^−7^ m GnRH for the indicated period (1–60 min) before termination of this single pulse by washing to remove the GnRH. They were then maintained in culture for a further for 300–359 min (*i.e.* the total incubation time was 6 h for all pulse widths) before luciferase measurements. Alternatively, the cells were treated as above except that the GnRH pulse was terminated by the addition of the PD184352 MEK inhibitor after 1–60 min. The *inset* shows raw Egr1-luc values from a representative experiment in which the transcriptional activity in the washed cells was higher than that in the PD184352-treated cells but only at the earlier time points. The ratio of luciferase activity in PD184352-treated cells to that in washed cells was also calculated, and the main figure shows data pooled from four such experiments (mean ± S.E., *n* = 4). This ratio is <1 for short pulses (indicating a greater transcriptional effect for short pulses terminated by washing) and approaches 1 (*horizontal dotted bar*, the value predicted if the termination method does not influence the transcriptional response) with higher pulse widths.

To test for functional relevance of the ERK activity continuing beyond the GnRH pulse, we compared transcriptional responses when stimulation was terminated by MEK inhibition or washing. The Fig. 7 *inset* shows data from an experiment in which cells received a single pulse of GnRH that was terminated by washing or by PD184352 addition and were then kept in culture for a further 6 h before Egr1-luc measurement. As expected, the transcriptional effect increased with increasing GnRH pulse width (irrespective of the termination method), and interestingly, the responses to short GnRH pulses (1, 5, and 15 min) were lower when the pulse was terminated by PD184352, whereas no such distinction was seen with longer GnRH pulses. To pool data from repeat experiments we divided the response with PD184352 termination by that with wash termination, reasoning that this would be 1 if the PD184352-sensitive post GnRH pulse was not functionally relevant. As shown, this value was <0.5 for the shortest GnRH pulses and approached 1 with a 60-min GnRH pulse (Fig. 7). Because this protocol influences ERK activity only after the GnRH pulse, the data reveal that the post-GnRH pulse ERK (and MEK) activity is indeed functionally relevant but only for the shorter pulses. This is presumably because the contribution of ERK activity in the minutes immediately after the pulse is significant for short pulses but is vanishingly small (compared with the effect driven by ppERK elevation during the pulse) for longer pulses. Collectively, these data support the idea that ERK activation continuing after the pulse underlies ERK-driven transcription with short GnRH pulses and could, therefore, explain the system insensitivity to pulse width (Fig. 2*A*).

## DISCUSSION

Cells respond to chemicals in their extracellular and intracellular environments, and information can be encoded not only in the chemical nature of the signal but also by signal dynamics (26). This is seen in the ERK signaling cascade where the dynamics of chemical stimulation determine the dynamics of ERK activation (16, 27), which then determines the pathway effects on cell fate (26, 32). When we used ERK2-GFP nuclear translocation as a live cell readout for ERK activation and Egr1-luciferase activity as a transcriptional readout, we found near linear relationships between GnRH pulse frequency and system output (Fig. 1; see also Ref. 16). These data are compatible with a simple scenario in which the system is linear and response dynamics are relatively fast (compared with the signal dynamics) such that integrated system outputs are roughly proportional to integrated input. In this case the system is expected to be equally sensitive to changes in pulse amplitude, frequency, and width (the three simplest means we have of controlling input integral), but this proved not to be the case. First, the Michaelis-Menten kinetics controlling receptor occupancy is sufficient to ensure that the system is more sensitive to changes in receptor occupancy than it is to changes in GnRH concentration (Fig. 1). Second, we find that the system is more robust to changes in pulse width than it is to changes in pulse frequency. This is most evident with the Egr1-luciferase data, where output is doubled by doubling pulse frequency, but a comparable increase in output requires a 10-fold increase in pulse width (Fig. 2).

To explore this further we fitted an ODE-based model of GnRH signaling to our data (“Experimental Procedures” and Table 1) and used it to predict dynamical responses to GnRH pulses (Fig. 3, *H–U*) and relationships between integrated HR and system output (Fig. 4). This revealed a characteristic feature of signaling cascades, that upstream responses often have rapid onset and offset and, therefore, mirror the dynamics of input closely (compare Fig. 3, *B* and *C* with *A*), whereas slowing of dynamics through the cascade leads to much slower responses downstream, and we find that this is associated with insensitivity to pulse width. Thus, the frequency-response curves reveal comparable sensitivity to pulse width and frequency at upper tiers and increasing robustness to pulse width moving down the cascade (Fig. 3).

We next considered why the system should be so effectively stimulated by short (1 min) GnRH pulses. One possibility is that a rapid and transient ppERK response favors signaling in the first minute, but this is not the case. Indeed, the effect of GnRH on ppERK levels was not maximal until 5–10 min, and the integrated ppERK2 response in the first minute was <2% of the response to 10 min (Fig. 2 and data not shown). An alternative possibility is that it reflects ERK activation beyond the GnRH pulse. In a *C. elegans* model, 20-s pulses of reduced NaCl concentration caused correspondingly brief pulses of Ca^2+^ elevation and slower MAPK activation that did not peak until ∼3 min after termination of the input. Although model architectures differ (notably, GnRHR-mediated ERK activation is not mediated by elevation of Ca^2+^) and response kinetics are different (pulses of sec-min as opposed to min-h herein), the earlier study also showed sensitivity to pulse frequency and relative insensitivity to pulse width (27), so we sought evidence for similar behavior with GnRH signaling. Using our mathematical model to predict ppERK responses to a 1- or 10-min pulse of GnRH revealed that ppERK levels would indeed outlast the hormone pulse, and wet laboratory measurements of ppERK levels confirmed this prediction. Indeed, with 1-min GnRH pulses (terminated by washing), at least 90% of the integrated ppERK response was predicted (Fig. 5*A*) or measured (Fig. 5*C*) to occur after the GnRH pulse. Similar data were found with GnRHR expressed in HeLa cells and with endogenous GnRHR of LβT2 cells (compare Figs. 5 and 6), and these data are remarkably similar to those reported for brief activation in *C. elegans* (27). The implication is that slow dynamics in activation and inactivation upstream cause ERK activity beyond the pulse, and this was confirmed by showing that when the activating pulse is terminated by MEK inhibition, the ppERK response returns much more rapidly to control (Figs. 5 and 6). Thus, ERK activity continues beyond the GnRH pulse because MEK remains active beyond the pulse.

The fact that most ERK activation occurs after a brief (1 min) pulse of GnRH raised the question of whether this post-GnRH activity is functionally relevant, and the sensitivity of the Egr1-luciferase reporter system enabled us to test this. We were able to stimulate the cells with a single short pulse of GnRH (as little as 1 min) and measure increased Egr1-luciferase activity at 6 h. Terminating the GnRH pulse by washing or by MEK inhibition provided a test for functional relevance of ERK activity after the pulse (because PD184352 was added to end the pulse and could, therefore, only influence ERK activity after the pulse). In this way we found that ERK activity after the GnRH pulse contributes to the transcriptional effect of short (1–15 min) but not long (30–60 min) GnRH pulses (Fig. 7). Presumably the contribution of ERK activity in the minutes immediately after the pulse is small (compared with the effect driven by ppERK elevation during the pulse) for longer pulses. These data, therefore, support the idea that ERK activation continuing after the pulse underlies ERK-driven transcription with short GnRH pulses and could, therefore, explain system insensitivity to pulse width at both the ppERK (Fig. 4*C*) and transcriptional (Fig. 2*A*) levels.

Although GnRH dynamics have long been known to be important for regulation of reproduction (8), our data provide more insight into GnRHR-mediated ERK activity specifically. Because this is essential for control of mammalian reproduction by GnRH pulses (2, 19), it is important to consider the data in a physiological context. GnRH secretory pulses can be inferred from electrical activity of hypothalamic GnRH neurons (multiunit activity), from peripheral LH pulses, and by microdialysis measurements of GnRH in the hypothalamo-hypophyseal portal circulation (33–38). Such studies reveal that GnRH pulse intervals vary widely in different physiological conditions and models, ranging from ∼15 min (in mice) to ∼16 h in pre-pubertal, postmenopausal, or luteal phase women (11, 12, 22, 39, 40). Measurement of GnRH concentration in the hypothalamo-pituitary portal circulation has been achieved in very few species, although early work revealed that GnRH pulses of goats approximate square waves with average width of 5 min (34), and this is why 5-min square wave stimuli were initially used here (Fig. 1). Pulse amplitude also varies, as illustrated by a 5-fold reduction in GnRH pulse amplitude from the luteal to the follicular phase (33). Early work also revealed a large variation in pulse amplitude, with ∼10-fold differences in maximal rate of GnRH release in consecutive pulses within an individual animal (35). GnRHR number also varies, changing through the oestrus cycle, pregnancy, and lactation and throughout development and aging (41, 42). In general, there is a dynamic range of ∼2–4-fold in GnRHR number. Together these data suggest a system in which sensitivity to pulse frequency and receptor occupancy provides the rationale for physiological regulation by control of GnRH pulse frequency and receptor number, whereas relative robustness to pulse amplitude could ensure gonadotropin release despite variation in maximal GnRH concentration from pulse to pulse.

Relatively few studies have addressed pulse width, but GnRH pulses in portal circulation of sheep and goats last for ∼5–10 min (33–35), and the duration of multiunit activity bursts is regulable, being increased from ∼2 to 10 min by ovariectomy in rhesus monkeys (36–38). A caveat here is that multiunit activity is not a direct measure of electrical activity in GnRH-secreting neurones and may well primarily reflect neuronal input to them. Nevertheless, our data suggest that any such variation in portal circulation GnRH pulse width would be unlikely to have a major influence on GnRHR-mediated ERK activity or gene expression. Here, it is important to note that relative insensitivity to pulse width develops working down the signal transduction pathway. For rapid upstream effects, sensitivity to pulse width and frequency is comparable. The obvious implication is that the rapid effects of GnRH on exocytotic gonadotropin secretion would be more sensitive to pulse width than the slower effects of GnRH on gene expression, such that regulation of pulse width provides a potential mechanism for differential regulation of these responses.

Overall, we illustrate here the mathematical underpinnings of a dynamical system that is robust to changes in pulse amplitude and width but is sensitive to changes in receptor occupancy and frequency, precisely the features that are regulated to exert physiological control of reproduction by GnRH *in vivo*. The relative insensitivity to pulse width increases as a consequence of slowing of responses working down the signal transduction cascade, and because this is characteristic of numerous signaling pathways, the behavior described may be generally applicable. Thus, we show that the information conveyed by different features of the dynamical input (frequency *versus* width) could be dictated by response kinetics and the response pathway locus under consideration.

## Footnotes

3 The abbreviations used are:

GnRH: gonadotropin-releasing hormone
GnRHR: GnRH receptor
LH: luteinizing hormone
FSH: follicle-stimulating hormone
BFP: blue fluorescent protein
ERK: we use the term ERK to mean ERK1 and/or ERK2, giving the numerical designation when a specific form is meant (as in ERK2-GFP)
MEK: MAPK/ERK kinase
NFAT: nuclear factor of activated T-cells
NLS: nuclear localization sequence
Ad: adenovirus
N:C: nuclear to cytoplasmic ratio
TF: transcription factor
TF1DT: TF1-dependent transcript
HR: hormone-bound receptor
ODE: ordinary differential equation
Egr-1: early growth response factor 1.
